# The study of Dominican amber-bearing sediments from Siete Cañadas and La Cumbre with a discussion on their origin

**DOI:** 10.1038/s41598-021-96520-3

**Published:** 2021-09-17

**Authors:** Paweł Stach, Lucyna Natkaniec-Nowak, Magdalena Dumańska-Słowik, Paweł Kosakowski, Beata Naglik, Przemysław Drzewicz, Jacek Misiak, Jaroslav Pršek, Carlos George, Ramón Elías Ramírez Gómez

**Affiliations:** 1 Faculty of Geology, Geophysics and Environmental Protection, AGH University of Science and Technology, 30 Mickiewicza Av., 30-059 Kraków, Poland; 2 Polish Geological Institute-National Research Institute, Upper Silesian Branch, 1 Królowej Jadwigi Str., 41-200 Sosnowiec, Poland; 3 Polish Geological Institute-National Research Institute, 4 Rakowiecka Str., 00-975 Warsaw, Poland; 4 Edificio Gubernamental Presidente Antonio Guzmán (Huacalito), Av. Estrella Sadhala, Santiago De Los Caballeros, 51000 Dominican Republic; 5 Residencial Rosmil, Los restauradores Calle 3 casa #7, Altos Distrito Nacional, Dominican Republic

**Keywords:** Geochemistry, Mineralogy, Petrology

## Abstract

The paper presents comprehensive mineralogical and geochemical characteristics of Dominican amber-bearing sediments from Siete Cañadas, Hato Mayor Province of the Eastern Mining District (EMD) in the Cordillera Oriental. The characteristics of rocks collected from the borehole in Siete Cañadas area (EMD) were compared with petrography of coaly shales from La Cumbre in the Northern Mining District (NMD). The mineralogy of the rocks was determined using transmitted and reflected light microscopy, scanning electron microscopy, Powder X-ray diffraction and Fourier Transform Raman Spectroscopy. Biomarker analyses by the gas chromatography–mass spectrometry were used to trace the genetic source and transformation stage of organic matter hosted in the core sediments. In this study, the characteristics of rocks from La Cumbre were supplemented with the petrographic data from our studies reported earlier. Based on the findings, it has been concluded that the basins in the investigated parts of the EMD and NMD regions were likely characterized by different, isolated palaeosettings. Transformation and maturation of terrigenous material were affected by locally occurring physicochemical conditions. In both amber deposits, the sedimentation of clastic and organic material proceeded in the presence of marine conditions. In case of the La Cumbre deposit (NMD area), the sedimentation underwent probably in the conditions of the lagoon environment, a shallow maritime lake or periodically flooded plain that facilitated organic matter decomposition and carbonation from meta-lignite to sub-bituminous coal (random reflectance of coal—R_r_^o^ = 0.39%). In the Siete Cañadas (EMD region), the sedimentation took place in a shallow saltwater basin, where terrigenous material was likely mixed with material found in situ (fauna fossils, carbonate-group minerals) to form the mudstones enriched in bituminous substance of low maturity. The organic matter found in the rocks from both deposits is of mixed terrestrial/marine origin and was deposited in the presence of low oxygen concentration and reducing and/or dysoxic conditions.

## Introduction

Fossil resins of various ages are found in many places around the world. The number of publications on these specific bituminous substances originated from the caustobiolite group is constantly increasing in every year^1–13^. Over a hundred of different fossil resins have been described, so far^14^. The term “amber” is used commonly as a synonym for fossil resin characterized by maturation grade, of different provenance, and geological and palaeobotanical source. Another term used by coal geologists for fossil resins is ‘resinite’, which is a microscopic material recognized as one of macerals—coal components. The oldest resinites of the Carboniferous age, were found within carbon sediments in Spain, France, Germany and Poland^15–20^. The youngest are of Holocene, identified in numerous deposits, among others in South America, Africa, Australia and New Zealand^21,22^.

Regardless of their age, all resins are products of vital activity of conifers and deciduous trees, gradually altered under the influence of palaeoenvironmental factors, i.e. climate, geological and biological conditions, that affected their fossilization. Hence, the chemical composition of fossil resins depends not only on botanical source but also specific conditions accompanying their formation, diastrophic processes (e.g. volcanism), climate changes, microbiological activity, and an interaction with surrounding rocks and sediments. These specific conditions affect significantly both molecular and isotopic composition of the resins. Nowadays, application of advanced analytical methods allows to elucidate and reconstruct the most of the fossil resin formation processes occurring from buried to present form^23^.

Among the worldwide fossil resin occurrences, the Dominican amber deposits are considered to be one of the largest^24–27^. They originated from deciduous trees similar to Acacia (*Hymenaea* genus), but their age is still in a dispute, and is estimated as middle Miocene 15–20 Ma^25,28,29^, 15.75–12.58 Ma^30^, or Pliocene–early Pleistocene^31^. The genesis of unusual accumulation of amber and lignitic deposits in Dominican Republic is still disputable as well. However, formation of large amber deposits was rather induced by serendipitous combination of several specific environmental conditions than a single environmental factor, for example like climate^25^.

**Figure 1:**
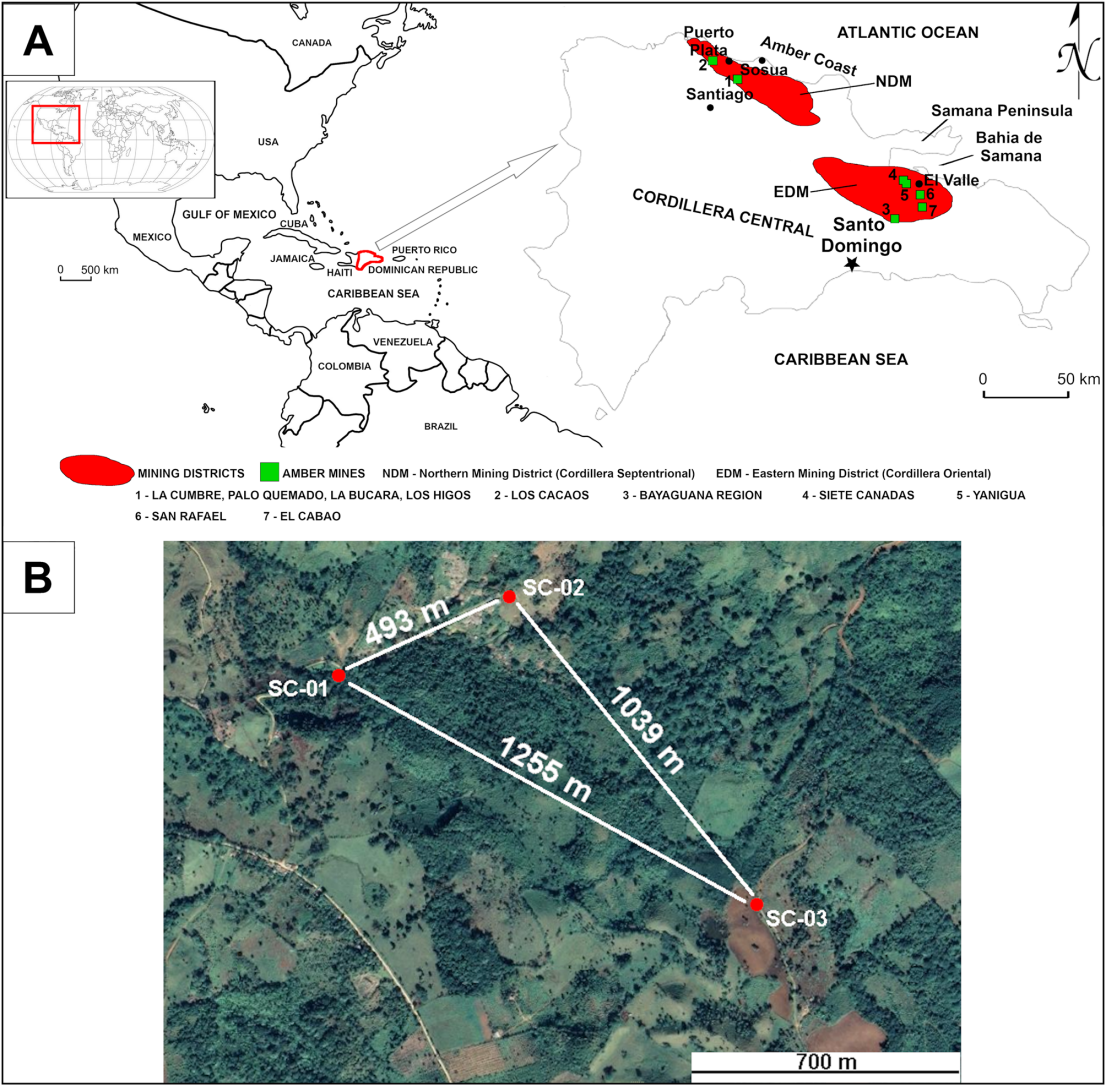
(**A**) Sketch map of the Dominican Republic with the location of the main amber deposits^10^. (**B**) Orthophoto map showing the orientation of the boreholes and distances between boreholes in the Siete Cañadas zone. (**A**) was drawn using CorelDRAW X5 and URL link is https://www.coreldraw.com/. (**B**) was generated using Google Earth 9.140.0.4 software and URL link is https://earth.google.com/.

Dominican amber deposits are mostly located in two regions, i.e. in the northwestern part of the country (the Cordillera Septentrional), near Santiago de los Caballeros (Northern Mining District; NMD), and the northeast of Santo Domingo in Cordillera Oriental (Eastern Mining District; EMD) (Fig. 1A). La Cumbre amber-bearing coaly shales (NMD) with fragments of coalified plant detritus of the La Toca Formation (LTF)^25,28,32,33^ were earlier characterized in detail^10^. Organic material in these rocks was in the transition stage from meta-lignite to subbituminous coal. Two types of resins were identified, i.e. a typical resinite that forms characteristic laminas, as well as detritic amber grains of different size and shape. It is assumed that both the flysch type and the amber-bearing sediments of the LTF were formed under deep-sea conditions^25,28,34–38^. However, the most recent reconstruction^10^ of the palaeoenvironment of the La Cumbre deposit (NMD) based on the facies analyses, mineralogical and petrographic studies of the amber-bearing sediments indicated that the transitional environment of marine sedimentation between a shallow maritime lake and periodically flooded plain cannot be excluded.

In this study, the results of petrogenetic investigation of the amber-bearing rocks from El Valle (Siete Cañadas area; EMD), collected from SC-02 borehole, are presented and discussed. The aim of the study was to elucidate depositional environment of the amberiferous rocks in this area based on host rocks mineralogy and geochemistry of associated organic matter. Novel data from scanning electron-microscopy (SEM–EDS), Fourier Transform-Raman spectroscopy (FT-RS), Powder X-ray diffraction (PXRD), Gas Chromatography–Mass Spectrometry (GC–MS) and Rock–Eval analysis were integrated into palaeogeographic chronology of depositional events and modelled burial history. Additional data on organic geochemistry of amber-bearing rocks from La Cumbre deposit (NMD) was obtained in order to complete and support their petrogenetic characterization reported in latest work, vide Stach et al.^10^. Finally, all data were used for comparison of amber-bearing sediments from NMD and EMD regions in terms of their petrogenesis and the palaeoenvironments. Based on the mineralogy of the rocks and the geochemistry of their organic matter, description of depositional environment of sediments, source area for their clastic components, and redox conditions were proposed and discussed for both regions of the Dominican Republic. Although the geology of Dominican Republic (Hispaniola Island) was relatively well described in literature^25,28,30–39^, there are still many gaps related to the geological processes that lead to formation of amber with unique daylight induced blue and green fluorescence. Here, we present new data related to fossil resin deposits in the Dominican Republic. Combining the previous^10^ and new results presented in this study, we discuss the geological processes and palaeoenvironment associated to the formation of Dominican amber deposits in Siete Cañadas and La Cumbre.

## Geological setting

The Eastern Mining District (EMD) is located northeast of Santo Domingo, on the northern border of Cordillera Oriental mountain range (Fig. 1A). Southern, western and eastern boundaries of this area are sedimentary (limestone, sandstone, greywacke, shale, conglomerate, breccia, etc.) and igneous Cretaceous rocks (gabbro, gabbrodiorite, tonalite). The Miocene formations in the EMD area submerge in the N-NE direction. The thickness of their Neogene cover varies from 100 m in the south to several hundred meters in the north^32,40,41,42^.

**Figure 2:**
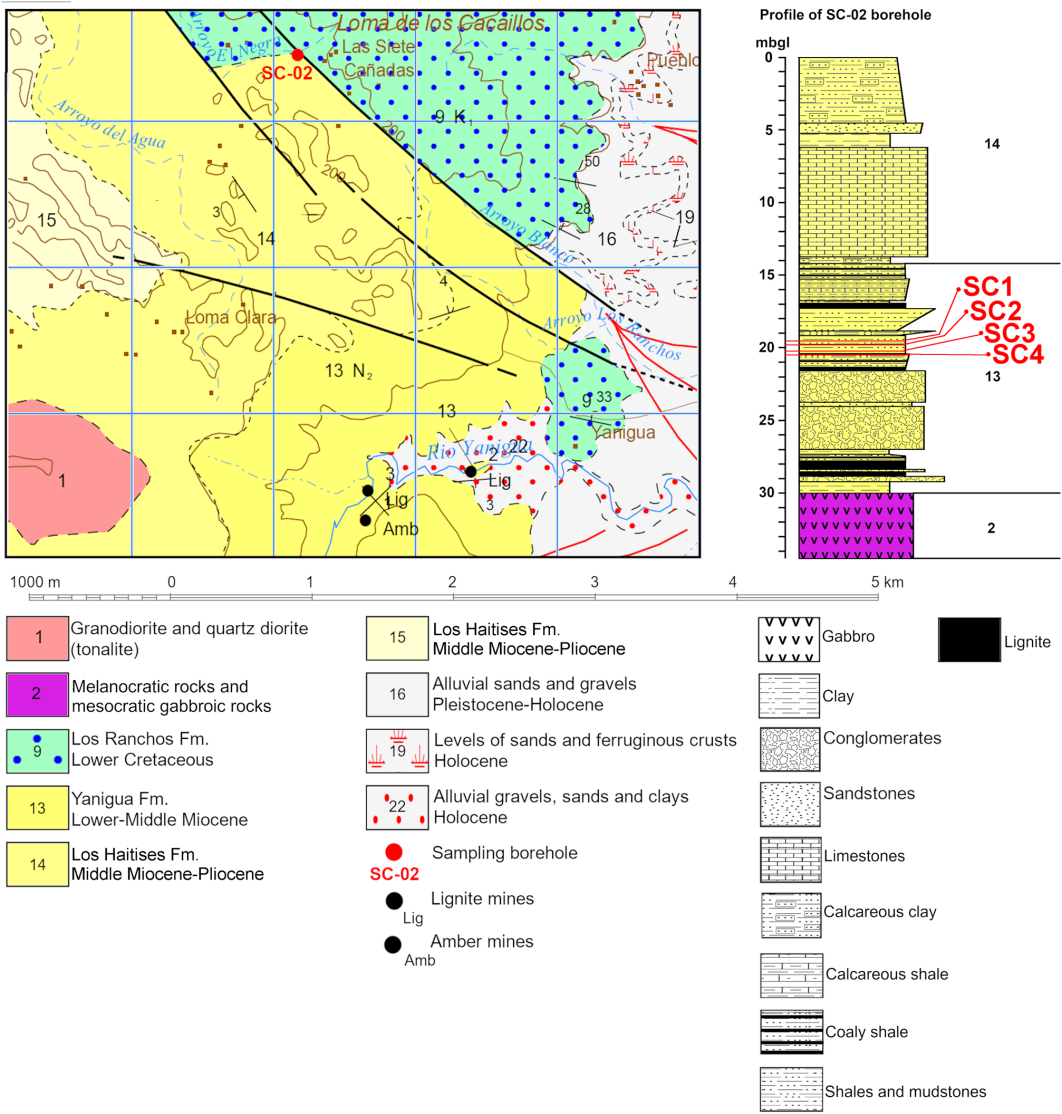
The geological map with the location and profile of sampling borehole SC-02 in the Siete Cañadas zone, based on the Geological Map of Dominican Republic at 1:50 000 scale—the El Valle sheet 6372-IV^43^ and the literature herein^25,38^. mbgl—metres below ground level; 9—Volcanic and metamorphosed (low grade) volcaniclastic rocks, 13—Alternation of clays, shales, sandstones and marls with coal layers and marly limestones, locally conglomerates, 14—Marly limestones and reef limestones, 15—Reef limestones. This figure was drawn using CorelDRAW X5 and URL link is https://www.coreldraw.com/.

In this region, the Miocene Basin rocks are represented by two complexes, i.e. Yanigua Formation (YF) (including the basal conglomerate) and Los Haitises Formation (LHF)^25^. The igneous rocks lie relatively close to the surface. Two of the three core drillings (SC-02 and SC-03), carried out in this region, reached gabbro at a depth of 34.50 and 37.20 m below ground level, respectively (Figs. 1B and 2).

According to available data, the YF complex is an amber-bearing unit, located around the current edge of the Neogene basin^42^. The thickness of this complex change from about 30 m (Siete Cañadas zone) to 40–60 m (Yanigua zone) in the north to even 100 m in the other places of the El Valle region. Basic conglomerate layers devoid of amber constitutes the footwall of the formation. Nature of the layers indicates a fluvial deposition environment. The other parts of the YF show rather slight lateral differences^25,40,41^. Dark clay and laminated sandy clays contain freshwater mollusks. The most commonly sediments are with lignite, clays, sandstones and limestones. The lamination of the sediments is usually parallel, sometimes with ripple marks visible in macro scale. Sandy clays are made of clay minerals, calcite, pyrite, limestone clasts, detritic quartz as well as lithoclasts of igneous rocks. They usually contain organic remnants of fresh and saltwater such as fossils of mollusks, ostracods, foraminifera, bryozoans, fish teeth, red algae, echinoids, and many others^26,27,30^. These fossils clearly indicate the significant impact of the upwelling process during the formation of the Yanigua Formation rock complex^30^. In these layers numerous irregular and flattened resin crumbs were found. They usually form the pockets or lenses, ranging from a few mm to several cm in size, or occur within the coal matrix with well-preserved plant remnants, suggesting local marshes and swamps. Biocalcarenites occur at the top of the YF (probably Cévicos Limestone), which turns into the LHF made up of limestones with plenty of fauna. Its thickness is about 300 m^25^.

Nowadays, in the EMD, within the YF amber is exploited in the vicinity of El Valle (Hato Mayor Province) in four mining zones, i.e.: Siete Cañadas, Yanigua, El Cabao and San Rafael (including Las Flores and Juan Bosch). In the borehole profiles from each of these zones, the layers of solid, black coloured lignite with metallic lustre form coal seams (Fig. 2).

The lignite layers mainly occur inside coaly and/or sandy clays, locally enriched in aggregates of gypsum. Amber is found within these lignite strata or 1.5–2.1 m below it. In the YF rocks, the amber crumbs were found in the drilling cores even at a depth of 82 m below the ground surface. Brouwer and Brouwer^32^ suggested that amber from that area was redeposited. On the other hand, Iturralde-Vinent^25^ did not corroborate the secondary origin of amber because of the shape of resin crumbs, i.e. sub-rounded, oval or stalactite-like which is characteristic for non-transferred specimens. The detailed geology of Northern Mining District was reported elsewhere^10^.

## Material and methods

The microscopic observations, and PXRD, FT-RS, GC–MS analyses were performed at the Faculty of Geology, Geophysics and Environmental Protection of AGH-University of Science and Technology in Krakow, Poland. The Rock–Eval analyses were made in the Oil and Gas Institute—National Research Institute in Krakow, Poland.

### Sampling

**Figure 3:**
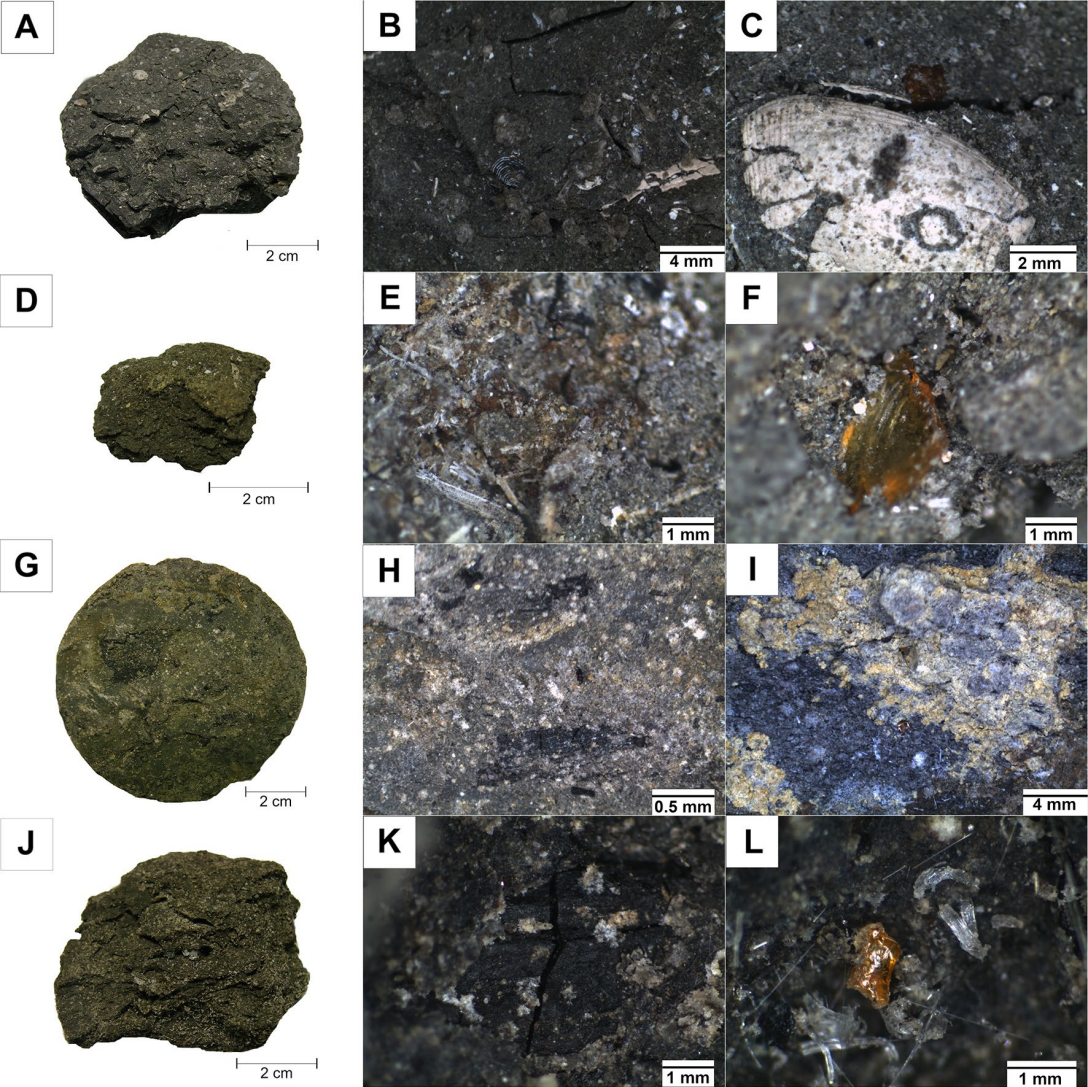
Macrophotographs of the studied rocks (**A**,**D**,**G**,**J**) and a stereoscopic microscope images (**B**,**C**,**E**,**F**,**H**,**I**,**K**,**L**). (**A**) Macrophoto of SC1 sample from the depth 19.5 mbgl; (**B**) stereoscopic microscope image of SC1 sample with fragments of carbonate shells; (**C**) carbonate shell (probably Ostracoda) and the fossil resin in the rock matrix (SC1 sample); (**D**) macrophoto of SC2 sample from the depth 19.85 mbgl; (**E**) needle-shaped crystals of gypsum in the rock matrix (SC2 sample); (**F**) fossil resin in the matrix (SC2 sample); (**G**) macrophoto of SC3 sample from the depth 20.25 mbgl; (**H**) lenses of carbonized plant detritus in the matrix (SC3); (**I**) the native sulfur on the matrix (SC3); (**J**) macrophoto of SC4 sample from the depth 20.5 mbgl; (**K**) fracture filled with coal in the rock matrix (SC4); (**L**) needle-shaped crystals of gypsum and fossil resin grain in the rock matrix (SC4).

The analytical material come from the Siete Cañadas area (SC-02 borehole, see Figs. 1A,B and 2) in Eastern Mining Districts of the Dominican Republic, where layers hosting amber reach up to 1.61 m of the thickness. The amber-bearing rock samples were taken from the cores repository in the Yanigua site, by the co-authors of this paper. They were collected from a depth of 19.5 m (SC1), 19.85 m (SC2), 20.25 m (SC3) and 20.5 m (SC4) below the ground level (Figs. 2 and 3A–L). Based on preliminary examinations, the SC-02 borehole was selected as the most representative in the studied area. Samples were taken from the amber-bearing zone of one of the sedimentary cycles immediately above the coal seam. The core was sampled continuously by taking a sectoral core section. For this purpose, half of the core was cut parallel to the axis.

For reference, the complementary tests, i.e. Powder X-ray diffraction, and the Rock–Eval pyrolysis supported by biomarkers analysis of coal shales from the La Cumbre deposit (NMD) have been also performed (samples LC1, LC2 and LC3). A petrography of these sediments has been recently published by Stach et al.^10^.

For geochemical analysis—Rock–Eval and GC–MS, the samples were cleaned and milled to a fraction below 0.2 mm.

### Stereoscopic microscope

Preliminary observations of the samples were made on the natural rock fractures with stereoscopic microscope SNZ-168, coupled with digital camera (with 0.75×, 1×, 2×, 3×, 4×, 5× objectives) and Panasis software. The polarized light (transmitted and reflected) microscopic observations of thin sections were made using two microscopes, i.e. the Olympus and Carl Zeiss Microscopy Primotech, the PZO and Axioplan (Zeiss-Opton). They were made to identify the mineralogy of the samples and characterize plant detritus.

### Scanning electron microscopy (SEM) coupled with energy dispersive spectroscopy (EDS)

SEM observations of fresh fractures of samples and polished sections were performed using the FEI Quanta 200 Field Emission Gun scanning electron microscope equipped with energy-dispersive X-ray spectrometer (EDS). The system operated at 20 kV accelerating voltage in a low-vacuum mode.

### Powder X-ray diffraction (PXRD)

PXRD analyses of rocks were carried out using a Rigaku SmartLab X-ray diffractometer (Neu-Isenburg, Tokyo, Japan) with the curved crystal graphite-monochromatized CuKα radiation*,* to identify phase composition of samples, especially type of clay minerals. The PXRD patterns were recorded in the range of 5°–75° 2Θ with step size 0.05°, counting time 1 s/step, operating voltage 45 kV and current 20 mA.

### Raman spectroscopy (RS)

Raman spectra were recorded on clean cleavage surfaces of the rock specimens using the Thermo Scientific DXR Raman microscope featuring 10×, 50× and 100× magnification objectives. The samples were excited with a 532-nm laser with power from 1 to 10 mW; exposure time was 3 s, the number of exposures was 10, and the laser focus diameter was approximately 2–1 µm. The spectra were corrected for background by the sextic polynomial method by the use of OMNIC 8.3.103 software. The Fourier Transform Raman microspectroscopy (FT-RS) analyses were performed mainly to identify the type of feldspars and carbonates and also the nature of the ore minerals.

### Microhardness testing

Measurement of microhardness of amber crumbs was made using a tester of Soviet production PMT-3. The testing procedure has been performed based on the Vickers methodology which involves pressing a quadrangular diamond pyramid with a dihedral angle equal to 136° on the flat surface of the tested material. The test was performed on the smooth surface of each 3 samples of Dominican amber, and repeating the measurement 30 times. The results obtained were afterwards statistically processed.

### The Rock–Eval pyrolysis method and biomarkers analysis

The Rock–Eval pyrolysis method and biomarkers analysis were applied in order to assess the quantity, kerogen type and thermal maturity of organic matter in the rocks. The samples from the Siete Cañadas area were analysed and compared to the specimens from the La Cumbre deposit performed to determine the genesis of their organic precursors and their depositional environment. The pyrolysis of rock samples was carried with the Rock–Eval Model 6 instrument according to Lafargue et al.^44^ and Behar et al.^45^. The pyrolytic apparatus was equipped with two ovens for pyrolysis and combustion processes with the programmed temperature. The temperature was conducted from 100 up to 850 °C. The hydrocarbons generated during analysis were measured by a flame ionization detector (FID), whereas the non-hydrocarbons compounds like CO_2_ and CO released during pyrolysis and oxidation stages were monitored by an infra-red detector (IR). The hydrocarbons detected by FID are represented by S_1_ and S_2_ peaks. Non-hydrocarbons compounds are produced during pyrolysis (up to 500 °C—S_3CO_ and up to 400 °C S_3CO2_ pyrolysis curves) and during oxidation (S_4CO_ and S_4CO2_ oxidation curves)^44^. The Rock–Eval 6 apparatus also allowed for the determination of the mineral carbon content (MINC), described by the peak S_5_ and S_3MINC_^44^. Based on these results, the parameters of the quality of the source rock were calculated: (1) organic carbon content (sum of S_1_, S_2_, S_3_CO, and S_3_CO_2_ peaks – all released during pyrolysis), (2) residual carbon RC (sum of the S_4_CO and S_4_CO_2_ peaks – obtained during the oxidation), (3) the oxygen index OI, (4) hydrogen index HI, and (5) temperature T_max_^44,45^.

Prior to the biomarkers analysis, the compounds were extracted with dichloromethane:methanol (93:7 v/v) in Soxhlet apparatus. The asphaltene fraction was precipitated with *n*-hexane. The remaining maltenes were then separated into compositional fractions of aliphatic hydrocarbons, aromatic hydrocarbons and resins by the use of column chromatography, using an alumina:silica gel (2:1, v/v) column (0.8 × 25 cm). The fractions were eluted with *n*-hexane, toluene, and toluene:methanol (1:1, v/v), respectively.

The isolated saturated hydrocarbon fractions were diluted in isooctane spiked with 5β-cholane and analyzed using gas chromatography-mass spectrometry (GC–MS). The analysis was carried out with an Agilent 7890A gas chromatograph (GC) equipped with an Agilent 7683B automatic sampler, an on–column injection chamber, and a fused silica capillary column (60 m × 0.25 mm i.d.) coated with 95% methyl/5% phenylsilicone phase (DB-5 ms, film thickness 0.25 μm). Helium was used as a carrier gas. The GC oven was programmed as follows: a temperature of 80 °C was maintained for 5 min, then it was ramped to 120 °C at the rate of 20 °C/min, after that, to 180 °C at the rate of 2 °C/min, and finally, it was ramped to 300 °C at the rate of 3 °C/min. The oven was kept at 300 °C for 35 min. The GC was coupled with an Agilent 5975C mass selective detector (MSD), which operated at an ion source temperature of 230 °C, ionisation energy of 70 eV, and cycle time of 1 s in a mass range from 45 to 550 Daltons. The aromatic hydrocarbon fractions were diluted in toluene and analyzed by GC–MS using the same equipment as for the saturated hydrocarbon fraction. Ortho-terphenyl was used as an internal standard. The GC oven was programmed as follows: a temperature of 80 °C was maintained for 1 min, then it was ramped to 120 °C at the rate of 20 °C/min, and after that, to 180 °C at the rate of 2 °C/min, and finally, was ramped to 300 °C at the rate of 3 °C/min. The oven was kept at 300 °C for 35 min. The MSD was operated with a cycle time of 1 s in a mass range from 45 to 550 Daltons.

## Results

### Mineralogy and petrography of amber-bearing rocks from the Siete Cañadas (EMD)

Fine-grained and poorly oriented, laminated texture was observed in investigated rock samples (Fig. 3A–L). Locally, they host differently coloured, fine crumbs of amber (Fig. 3F,L) and fauna fossils remnants such as shells of mollusks or/and ostracods, corals, with sizes up to 1 cm (Fig. 3B,C). They are particularly abundant in the specimens collected from the upper part of the profile (SC1, SC2).

**Figure 4:**
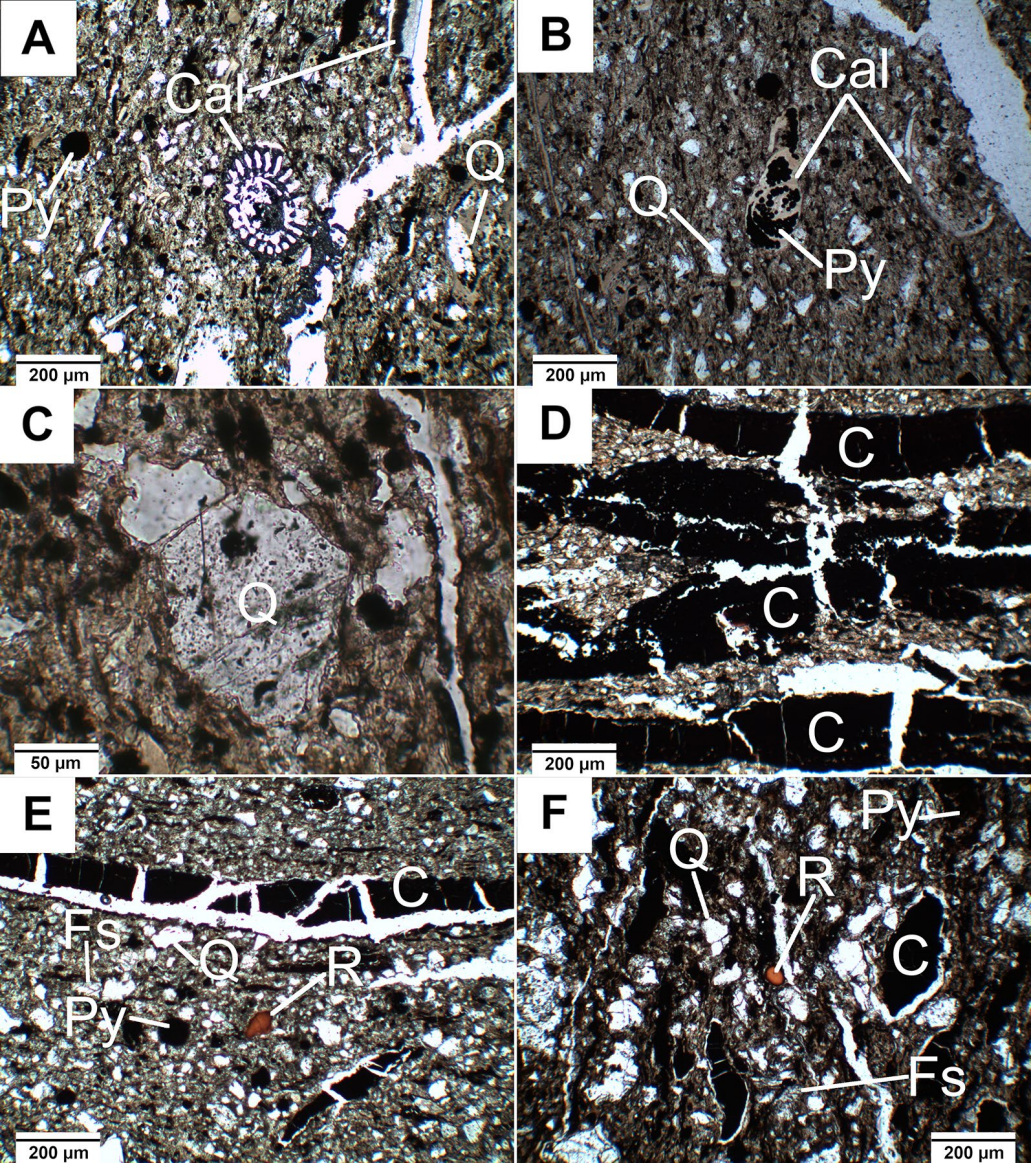
Microphotographs of the studied rocks. (**A**) Quartz (Q) and opaque minerals (mainly pyrite—Py) with a carbonate fragment of a coral and shells (Cal) in the matrix (SC1 sample, NX); (**B**) grain components of the rock with partially pyritized (Py) shell fragments (SC1 sample, 1N); (**C**) quartz grains with an euhedral shape (SC1 sample, 1N); (**D**) cracked coal laminae in the matrix (SC3 sample, NX); (**E**) quartz (Q), feldspars (Fs), opaque minerals (mainly pyrite—Py), cracked coal laminae and lenses (C) with fossil resin crumb (R) (SC3 sample, NX); (**F**) quartz (Q), feldspars (Fs), opaque minerals (mainly pyrite—Py), cracked coal lenses (C) with fossil resin crumb (R) in the rock matrix (SC4 sample, NX).

The pelitic-aleuritic and parallel rock texture characteristic of mudstone, was better marked by polarized, transmitted light (Fig. 4A–F). Generally, a slight increase in grain size of clastic material in the direction from the top (100 µm) to the bottom (over 200 µm) of the profile is observed. Similarly, the content of organic material forming thin laminae in clay-clastic matrix is also gradually changing with depth; it is higher in specimens coming from the bottom of the profile. The average composition of the rocks includes clay minerals (44 vol.%), quartz (20 vol.%), feldspars (9 vol.%), gypsum (1 vol.%) and opaque, ore minerals, mainly represented by framboidal pyrite (15 vol.%).

**Figure 5:**
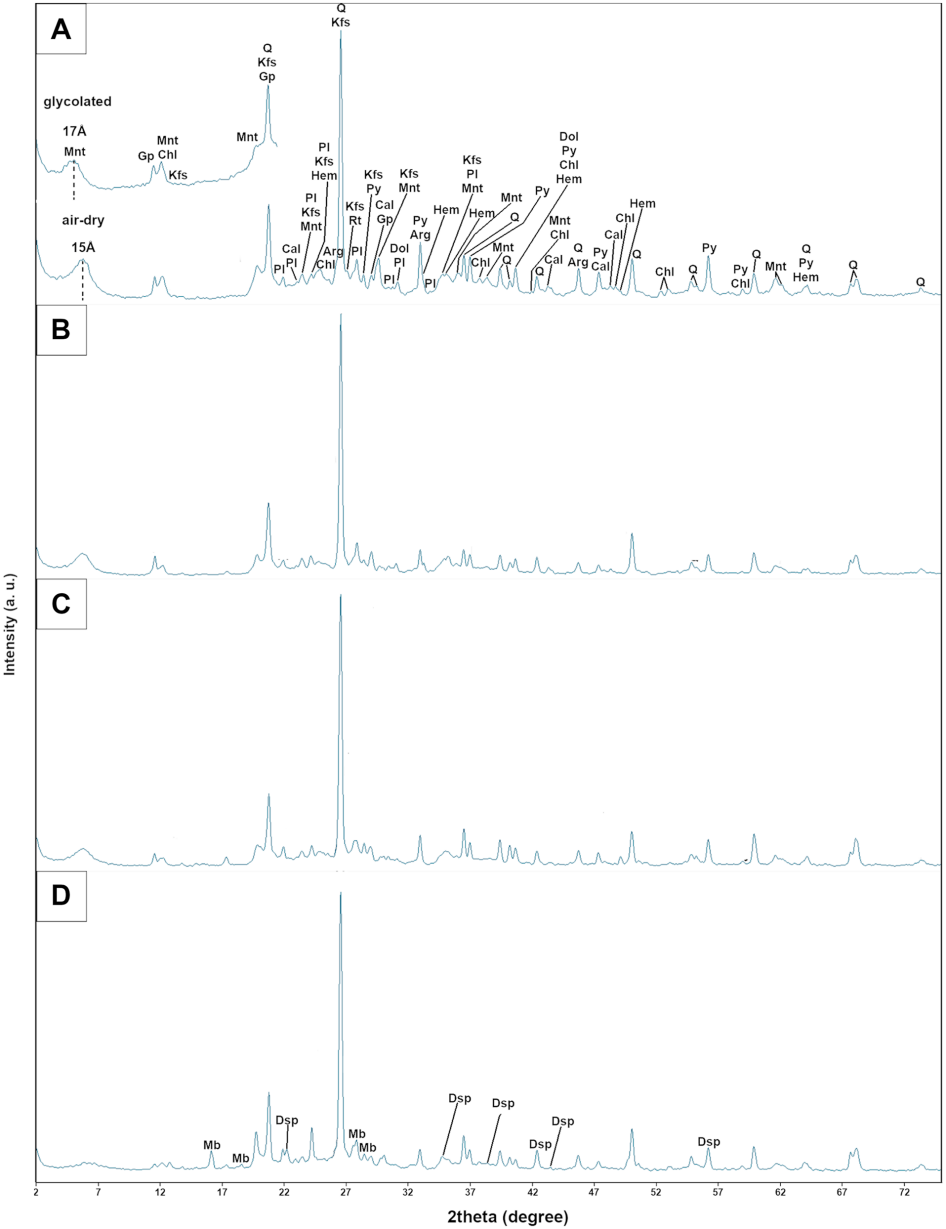
X-ray diffraction of studied rocks: (**A**) reflections from glycolated saturation and from air-dry preparations (SC1); (**B**) SC2, (**C**) SC3, (**D**) SC4. Symbols: Arg—aragonite; Cal—calcite; Chl—chlorite; Dol—dolomite; Dsp—diaspore; Gp—gypsum; Hem—hematite; Kfs—K feldspar (microcline, orthoclase); Mb—mirabilite; Mnt—montmorillonite; Pl—plagioclase; Py—pyrite; Q—quartz; Rt—rutile.

Powder X-ray diffraction (PXRD) analyses (Fig. 5A–D) revealed the presence of quartz and feldspars (both K-feldspars and plagioclases) and also the carbonates, i.e. calcite, aragonite and dolomite, probably originated from calcareous fossils found in the rock matrix (SC3, SC4). Clay minerals are represented by montmorillonite, and chlorites, while ore-bearing minerals by pyrite, rutile, hematite and ilmenite. Slight variation of accessory components was noted in the specimen coming from the bottom part of the profile (SC4). Besides gypsum found in all rocks, it also contains mirabilite Na_2_[SO_4_]*10H_2_O and diaspore, an aluminium hydroxide group mineral.

**Figure 6:**
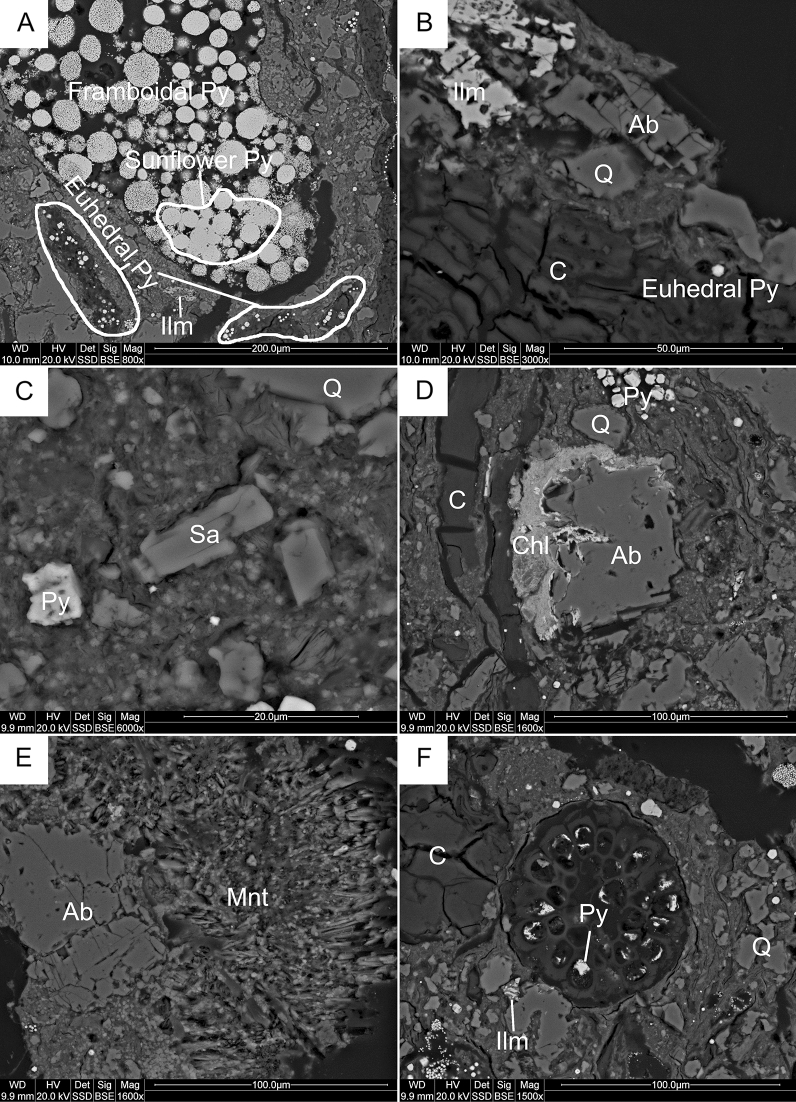
Thin-section scanning electron backscatter photomicrographs (SC4 sample). (**A**) The morphologies and arrangements of pyrite (Py) and iron–titanium oxides (Ilmenite—Ilm); (**B**) Albite (Ab) partially replaced by ilmenite (Ilm) and additionally euhedral pyrite (Py) grains and coalified plant detritus lamina; (**C**) Sanidine (Sa), detrital pyrite (Py) and quartz (Q) grains; (**D**) Partially chloritized (Chl) albite (Ab) grain with quartz (Q), euhedral pyrite (Py) and coalified plant detritus lamina; (**E**) Albite (Ab) partially replaced by Ca–Mg smectite (montmorillonite—Mnt); (**F**) The cross section of pollen with syngenetic pyrite (Py) inside cells.

Based on the microscopic observation, quartz forms various grains of euhedral habit with sharp edges, locally showing traces of magmatic corrosion, or sickle-shaped cracks characteristic of pyrogenic silica phase (Fig. 4B,C,E,F). Feldspars forming euhedral laths, are represented by sodium plagioclases (albite Ab_100-91_An_0-9_) and alkali feldspars (Or_54-60_Ab_46-40_) (Figs. 4E,F and 6B,C,E). They are strongly cracked and partially replaced by microcrystalline chlorite, Ca-Mg smectite, and Fe, Ti oxides/hydroxides (Fig. 6B,D,E). White mica (muscovite) forms single, fine plates randomly scattered within the rock matrix. Pyrite manifests its abundant presence in the rocks by three diagnostic bands at 432, 378, 343 cm^−1^ in the Raman spectra^46–48^. It forms at least three morphological types: (1) framboidal, (2) recrystallized and (3) euhedral crystals. It forms individual grain ranging in size from a few to several dozen micrometres (Fig. 6A–D,F) or aggregates of various shapes found in veins, coaly laminas, within plant detritus or randomly distributed in the rocks. Titanium minerals form euhedral or anhedral grains up to 100 μm in size with low reflection ability.

**Figure 7:**
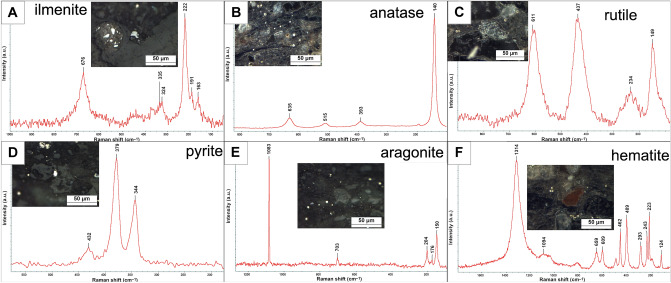
Raman spectra of the compounds of amber-bearing sediments. (**A**) Ilmenite (SC4 sample); (**B**) anatase (SC4 sample); (**C**) rutile (SC4 sample); (**D**) pyrite (SC1 sample); (**E**) aragonite (SC1 sample); (**F**) hematite (SC4 sample).

Fourier Transform Raman microspectroscopy (FT-RS) revealed the presence of Ti oxides such as rutile and anatase, and also Fe-Ti oxides (ilmenite) (Fig. 7A–C). The occurrence of ilmenite is indicated by characteristic bands at 222, 330, 368 and 683 cm^–1^ on the spectra^49,50^. The Raman bands at 149, 234, 437, and 611 cm^−1^ are assigned to rutile^51,52^. The presence of anatase corresponds to very intensive band at 140 cm^-1^ and less intense and broad lines at 393, 516 and 635 cm^−1^ ^53,54^. In general, the highest amounts of titanium oxides are found in the rocks from the lowest part of the profile (SC4). Conversely, pyrite is the most abundant in the rocks from the upper part of the profile (SC1, SC2; Fig. 7D). Occasionally, fine (25 μm in size) grains of REE-bearing phosphates (monazite) also occur in the rock matrix. The single anhedral crystals of strontium sulphates as well as acicular crystals of calcium sulphates fill fractures and cracks in the close vicinity of sodium feldspars.

The plant detritus form lenses and veins, strongly fractured, and occasionally filled with syngenetic crystals of pyrite. Locally, the traces of well-preserved cell structure of plant was observed on SEM images (Fig. 6F). The abundant plant detritus locally hosts the tiny grains of fossil resins (e.g. Fig. 4E,F) and abundant fragments of fauna fossils (e.g. Fig. 4A,B). The remnants of fauna fossils are composed of carbonate-group minerals. The characteristic bands at 1083, 710, 279 and 152 cm^−1^ on RS spectra indicate the presence of calcite in investigated samples while the bands at 1083, 703, 204, 176 and 150 cm^−1^ correspond to the presence of aragonite (Fig. 7E)^55–57^. Locally, the remnants of fauna fossils are altered by pyritization and silification (Fig. 4B).

Diffuse pigment coloured the rock matrix in red-brown. The bands at 223, 243, 293, 409, 495, 609 cm^−1^, and very intense at 1314 cm^−1^ correspond to hematite (Fig. 7F)^58–60^.

### Characteristics of amber from the Siete Cañadas (EMD)

Most of the fossil resins found in the rocks from the SC-02 borehole form tiny grains, up to a dozen millimetres in size, with conchoidal fracture and showing slightly rounded shapes (Fig. 3F,L). They are brown, orange or yellow in colour, sometimes cracked or crushed, and showing high brittleness.

For three resin crumbs separated from the rocks of Siete Cañadas area the microhardness tests were performed. The measured values (n = 30) fluctuated from 17.01 kgf/mm^2^ (Hv_min_) to 36.37 kgf/mm^2^ (Hv_max_), i.e. 166.81–356.67 MPa. The average value was 27.90 kgf/mm^2^ (273.61 MPa); SD = 5.05 kgf/mm^2^ (49.52 MPa). These values are very similar to that of fossil resins from La Cumbre deposit^10^. The resins have strong fluorescence from green–blue to blue in UVL (365 nm). Their fluorescence intensity is higher than those observed for fossil resins from La Cumbre deposit^10,61^.

### Quantitative composition of plant detritus from the Siete Cañadas (EMD)

**Figure 8:**
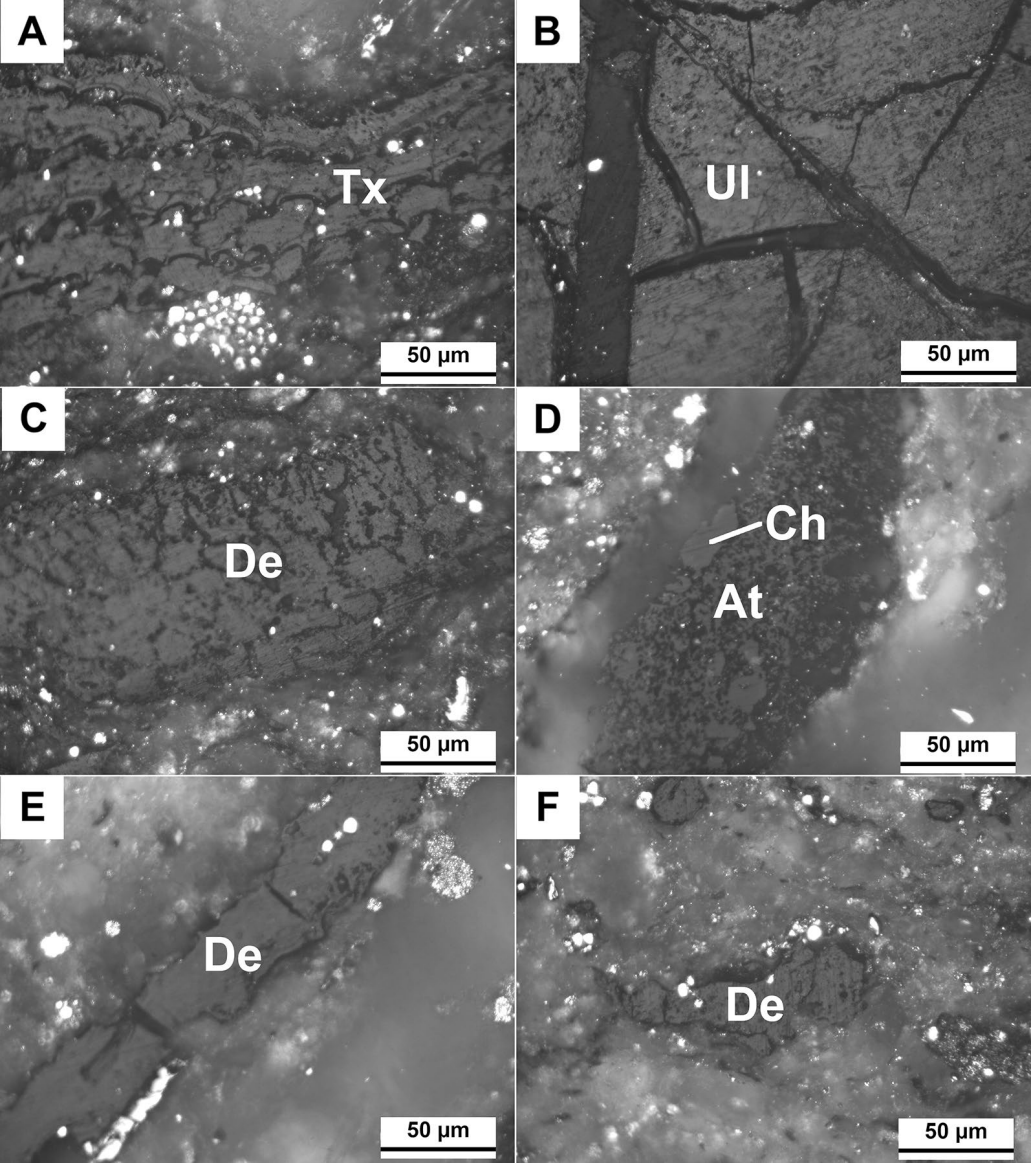
Microphotographs of shale with plant detritus under polarized reflected white light (oil immersion; SC4 sample). (**A**) Fragment of preserved wood with a visible cell structure—textinite (Tx); (**B**) Piece of wood with a damaged cell structure—ulminite (Ul). Visible cracks; (**C**) Accumulation of detritic plant—densinite (De); (**D**) Plant detritus—atrinite (At) with oval clusters of corpohuminite (Ch); (**E**) Laminae of detritic plant—densinite (De); (**F**) Plant detritus dispersed in the rock matrix—densinite (De).

The rock samples from profile SC-02 were identified as mudstones, strongly saturated with plant detritus (Fig. 8A–F) that were randomly dispersed in the rock matrix or formed thin layers (Fig. 8E). The size of individual plant remnants usually are in the range from a few µm to about 1 mm. They exhibit various shapes; from shaggy (detrital forms) (Fig. 8F) to oval. The latter remains fully preserved plant fragments (Fig. 8C,D) with well visible cellular structure (Fig. 8A). In other plant detritus, the original structure has been more or less disturbed (Fig. 8B). Occasionally, small crumbs of dark yellow fossil resin are found close to these plant fragments.

The plant detritus identified in the rocks belongs exclusively to the huminite group. These macerals only occasionally have well-preserved plant tissue. They usually have a detrital or gelified forms. Species represent all classification subgroups, i.e. humotelinite (textinite, ulminite), humodetrinite (atrinite, densinite) and humocolinite (corpohuminite, gelinite) (Fig. 8A–F). Textinite, the fragments of wood with well-preserved cellular structure, was found only locally (Fig. 8A). Ulminite is a maceral with more advanced transformations in its structure, which led to its complete destruction (Fig. 8B). The presence of atrinite and densinite (Fig. 8C,D) indicates that detritus is composed of herbaceous plants or fragment of trees. These macerals show differences in the degree of density of the detritus. In the atrinite, the material is loosened contrary to the densinite, where it is more dense. At the contact of densinite, some concentrations of an oval gel-like substance—corpohuminite are found (Fig. 8D).

### Results of Rock–Eval and bitumen extraction analysis for sediments from the Siete Cañadas (EMD) and La Cumbre (NMD)

**Figure 9:**
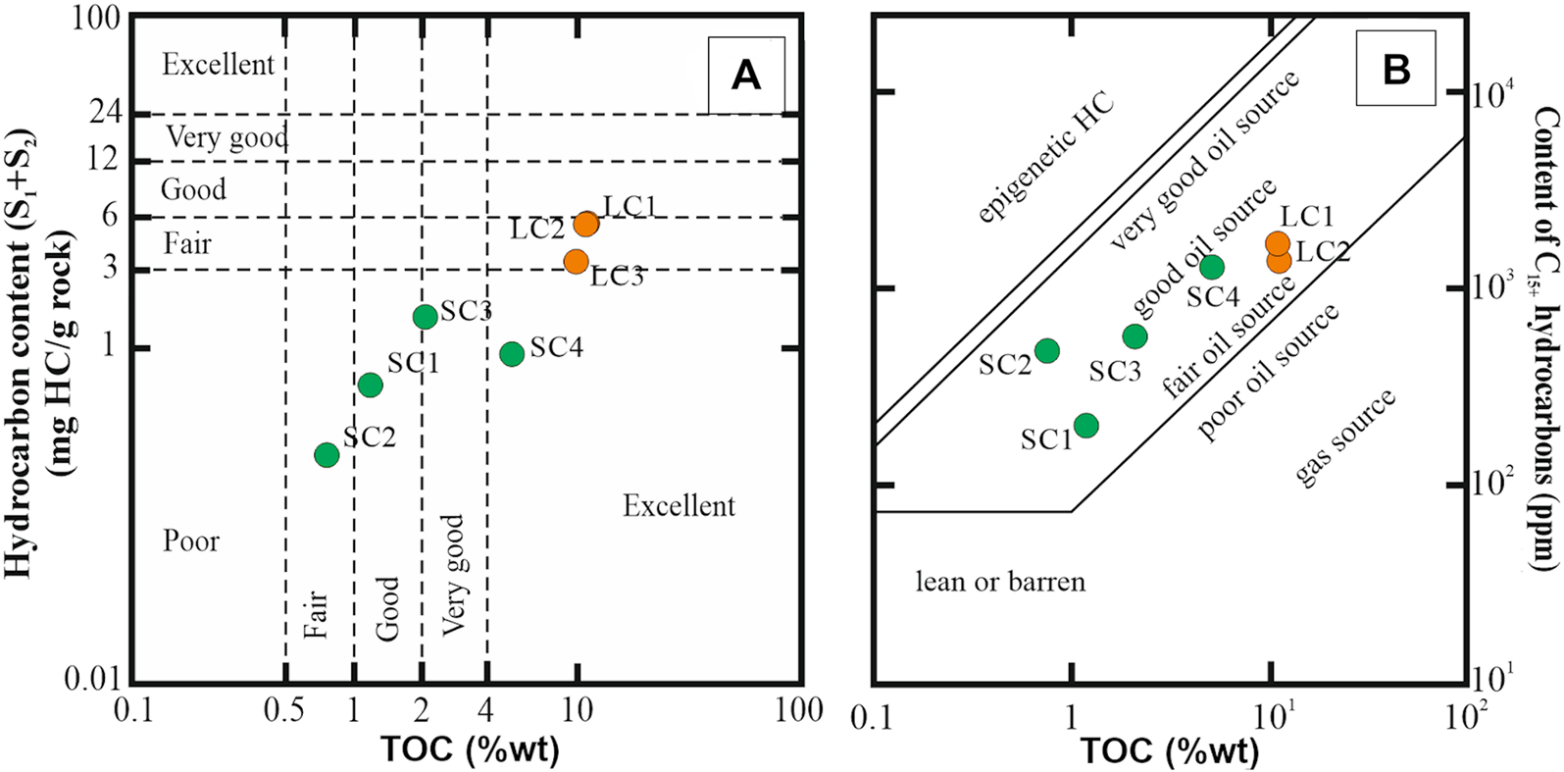
Petroleum source quality diagram for organic matter of La Toca and Yanigua formations. Petroleum quality classification after (**A**) Peters and Cassa^62^, (**B**) propose omit Hunt^63^ and Leenheer^64^.

In the rock samples from the Siete Cañadas (SC1-SC4), the total organic carbon content (TOC) varies between 0.75 to 5.11 wt.%, with an average value of 2.3 wt.% (Table 1). The hydrocarbon content (S_1_ + S_2_) ranges from 0.60 up to 1.53 mg/g of rock (Table 1, Fig. 9A) with an average of 0.8 mg/g of rock. This indicates that the hydrocarbon potential of analyzed rocks from this region varies from fair to excellent (Fig. 9A).

**Table 1:** Results of Rock–Eval pyrolysis.

Sample	TOC	T_max_	S_1_	S_2_	S_3_	PI	HI	OI	MINC	PC	RC
LC1	11.13	379	0.78	4.72	11.33	0.14	42	102	0.77	0.98	10.15
LC2	10.95	388	0.69	4.72	11.17	0.13	43	102	0.71	0.96	9.99
LC3	9.92	385	0.39	2.87	10.58	0.12	29	107	0.73	0.76	9.16
SC1	1.18	425	0.09	0.51	1.42	0.14	43	120	1.02	0.12	1.06
SC2	0.75	413	0.05	0.18	1.71	0.23	24	228	0.28	0.09	0.66
SC3	2.08	335	0.41	1.12	3.2	0.27	54	154	0.5	0.28	1.8
SC4	5.11	377	0.18	0.74	8.13	0.19	14	159	0.99	0.42	4.69

*TOC* total organic carbon, in wt.%; *T*_*max*_, temperature, in ^o^C; *S*_*1*_, free hydrocarbons, in mg/g rock; *S*_*2*_, heavy hydrocarbons, in mg/g rock; *S*_*3*_, CO_2_ content, in mg/g rock; *PI* productivity index; *HI* hydrocarbons index, in mg/g TOC; *OI* oxygen index, in mg/g TOC; *MINC* mineral carbon, in wt.%; *PC* pyrolytic carbon, in wt.%; *RC* residual carbon, in wt.%

The La Cumbre deposit was represented by three samples—LC1, LC2 and LC3. The TOC values in these samples are much higher, i.e. 9.92 to 11.13 wt.% (Table 1), with an average value of 10.7 wt.%. The hydrocarbon content values (S_1_ + S_2_) are also higher, from 3.26 to 5.50 mg/g rock, what might indicate that these rocks have excellent hydrocarbon potential (Fig. 9A).

Similarly, the high amount of bituminous extract, ranging from 565 to 3630 ppm in Siete Cañadas rocks, and 3906 and 4790 ppm in La Cumbre rocks (Table 2, Fig. 9B). In both areas results indicate the good hydrocarbon potential. The extract is dominated by resins and asphaltenes fractions, ranging from 64 to 86% in rocks from Siete Cañadas, and 83 and 86% in La Cumbre (Table 2). The proportions of saturated and aromatic hydrocarbon fractions in extract are smaller.

**Table 2:** Results of Soxhlet extraction and compositional fractions of bitumen.

Sample	Extract (ppm)	Fraction (%)				Sat./Aro.
		Sat.	Aro.	Res.	Asph.	
LC1	3906	4	11	8	78	0.4
LC2	4790	4	13	6	77	0.3
SC1	565	21	16	32	32	1.3
SC2	1361	9	12	21	59	0.8
SC3	1608	3	11	8	78	0.3
SC4	3630	3	18	9	69	0.2

*Sat.* saturated hydrocarbons, *Aro.* aromatic hydrocarbons, *Res.* Resins, *Asph.* asphaltenes.

### Biomarkers analysis for sediments from the Siete Cañadas (EMD) and La Cumbre (NMD)

The rocks from Siete Cañadas (SC1-SC4) and the La Cumbre (LC1, LC2) deposits contain few groups of compounds: *n*-alkanes, acyclic isoprenoids, terpanes, and steranes.

**Figure 10:**
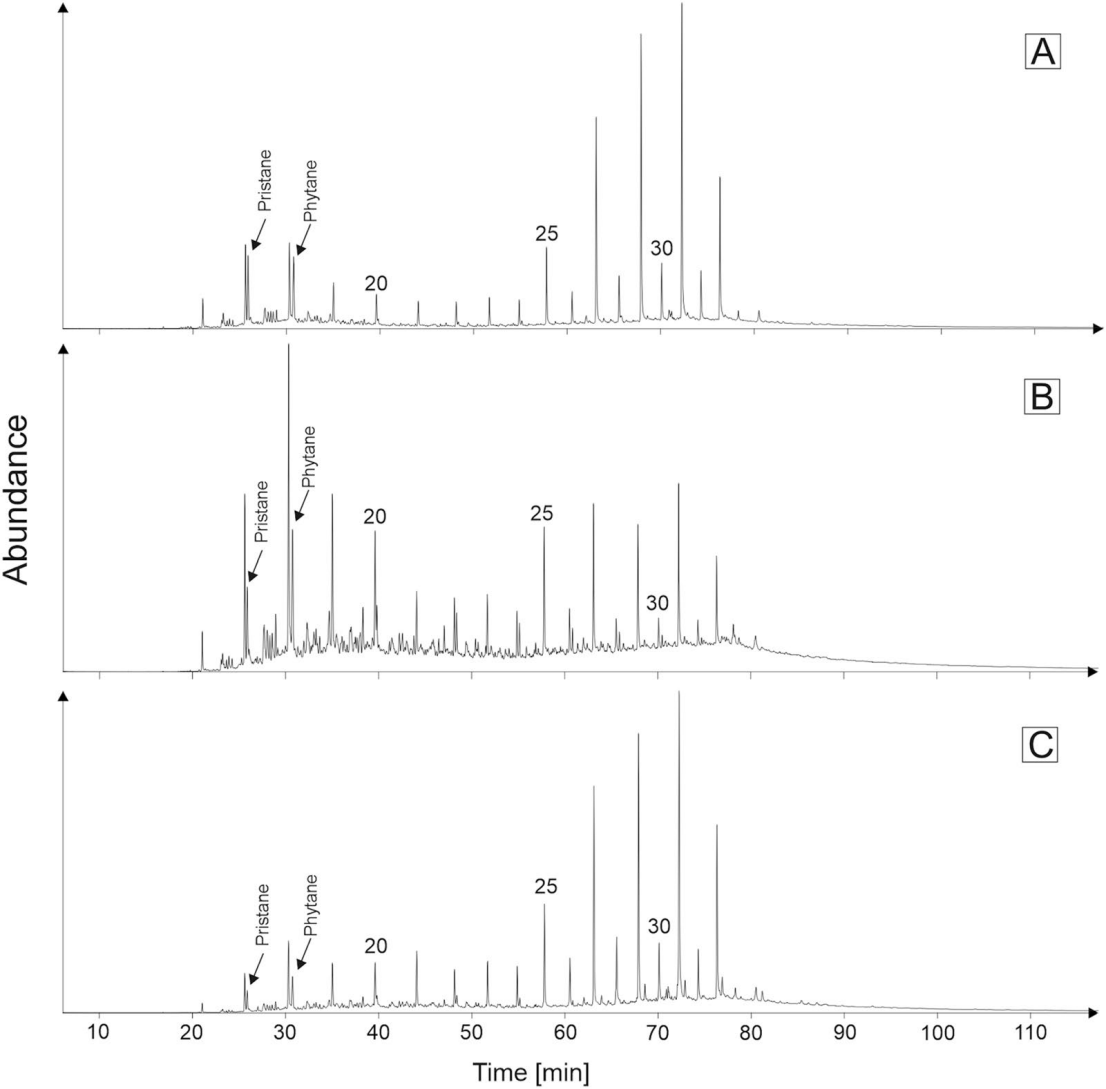
Mass chromatograms (*m/z* 71) saturated hydrocarbon fractions, (**A**) LC1 sample, (**B**) SC1 sample, and (**C**) SC4 sample. 20, icosane; 25, pentacosane; 30, triacontane.

The analysis of fragmentation ion *m/z* 71 revealed presence of *n*-alkanes from C_14_ to C_31_ homologues (Fig. 10). They exhibit monomodal distribution in samples from the La Cumbre deposit (Fig. 10A) with the domination role of short-chain compounds, and bimodal distribution with maximum of C_18_ and C_31_ in samples from the Siete Cañadas area (Fig. 10B,C). The Carbon Preference Index (CPI), calculated according to Kotarba et al.^65^, is in the range from 1.26 to 2.67 for mudstones from the Siete Cañadas, and in the range from 2.81 to 2.84 for coaly shales from the La Cumbre (Table 3). The calculated Terrigenous-Aquatic Ratio^66^ is higher than 1.0 for the samples from both regions and ranging from 1.34 to 8.56 in Siete Cañadas, and 6.05 and 7.04 in La Cumbre samples (Table 3).

**Table 3:** Biomarker indicators of origin of organic matter and palaeoenvironment conditions.

Sample	CPI_(Total)_	CPI_(17–23)_	CPI_(25–31)_	Pr/Ph	Pr/(Pr + Ph)	Pr/*n*-C_17_	Ph/*n*-C_18_	TAR_HC_	P_aq._
LC1	2.81	0.91	4.95	0.92	0.48	0.95	1.04	6.05	0.15
LC2	2.84	0.94	4.66	1.01	0.50	1.22	1.27	7.04	0.13
SC1	1.26	0.66	3.90	0.53	0.35	0.61	0.56	1.34	0.38
SC2	1.42	0.66	3.62	0.49	0.33	0.52	0.51	2.07	0.31
SC3	2.67	0.95	4.33	0.71	0.42	0.60	0.64	6.43	0.34
SC4	2.35	0.95	3.79	0.51	0.34	0.64	0.64	8.56	0.19

*CPI* Carbon Preference Index, *Pr* pristine, *Ph* phytane, *TAR*_*HC*_ Terrigenous-Aquatic Ratio, *P*_*aq.*_ terrestrial/aquatic plants in aquatic environments, counted as (*n*-C_23_ + *n*-C_25_)/(*n*-C_23_ + *n*-C_25_ + *n*-C_29_ + *n*-C_31_).

The acyclic isoprenoids—pristane (Pr) and phytane (Ph) were observed also on chromatograms recorded at m/z 71 (Fig. 10). The juxtaposition of Pr/*n*-C_17_ and Ph/*n*-C_18_ ratio was used to determining maturity and origin of organic matter. In the Siete Cañadas samples the values of Pr/Ph, Pr/(Pr + Ph), Pr/n-C_17_, and Ph/n-C_18_ ratios fall in the range from 0.49 to 0.71, 0.33–0.42, 0.52–0.64, and 0.51–0.64, respectively (Table 3). For the La Cumbre samples the values of these indices are higher and were as follow: Pr/Ph = 0.92 and 1.01, Pr/(Pr + Ph) = 0.48 and 0.50, Pr/n-C_17_ 0.95 and 1.22, and Pr/n-C_18_ 1.04 and 1.27 (Table 3).

Chromatograms of the sterane distribution were recorded at *m/z* 217 mass ion, and αββ steranes at *m/z* 218. The combined results of these single mass ion chromatograms allowed to calculate the regular ααα steranes distribution, C_27_diasteranes/(diasteranes + regular steranes), C_27_dia/(dia + reg), C_29_S/(S + R), and C_29_αββ/(ααα + αββ). The distribution of regular ααα steranes revealed that C_29_ regular steranes play a dominant role in all samples (Table 4). The results of C_27_dia/(dia + reg) ratio linked with palaeoenvironment conditions in all samples were 0.00 (Table 4). Linked with thermal maturity of organic matter, C_29_S/(S + R) and C_29_αββ /(ααα + αββ), were very low, in range from 0.01 to 0.08, and 0.03 to 0.24 respectively (Table 4).

**Table 4:** Biomarker indicators sources and maturity of organic matter.

Sample	% C_27_aaa20R	% C_28_aaa20R	% C_29_aaa20R	C_27_Dia/(Dia + Reg)	(C_21_ + C_22_)/(C_27_ + C_28_ + C_29_)	C_29_aaa20S/(S + R)	C_29_abb/(aaa + abb)	C_29_aaa20S/20R	%Tricyclic Terpanes	% Pentacyclic Terpanes	% Steranes	Tricyclic/ Pentacyclic Terpanes	Steranes/Terpanes
LC1	14.47	5.27	80.26	n.d	n.d	0.02	0.03	0.02	11.35	43.26	45.39	0.26	0.83
LC2	11.74	11.01	77.25	n.d	n.d	0.05	0.16	0.05	n.d	n.d	n.d	n.d	n.d
SC1	33.81	10.58	55.60	n.d	n.d	0.07	0.15	0.07	28.39	55.33	16.28	0.51	0.19
SC2	32.28	10.02	57.70	n.d	n.d	0.08	0.24	0.09	24.73	58.23	17.04	0.42	0.21
SC3	25.24	7.65	67.11	n.d	n.d	0.04	0.03	0.05	13.00	57.94	29.05	0.22	0.41
SC4	6.61	10.71	82.67	n.d	n.d	0.01	0.14	0.01	14.40	44.23	41.36	0.33	0.71

*Dia* diasteranes, *Reg* regular steranes, *n.d.* not detected.

Terpanes were observed on chromatograms recorded at m/z 191. The list of determined terpane compounds are given in the Table 4. For rocks from Siete Cañadas the juxtaposition of tricyclic/pentacyclic terpanes and steranes/terpanes ratio varies from 0.22 to 0.55, and 0.19 to 0.71 respectively (Table 4). In the sediments from the La Cumbre deposit the terpanes were identified only in one sample, tricyclic/pentacyclic terpanes and steranes/terpanes ratios were 0.26 and 0.83 respectively.

## Discussion

### Depositional environment and diagenesis processes of the amber-bearing rocks in Siete Cañadas area (EMD)

Stratigraphy, palaeontology and sedimentology of amber-bearing deposit and rocks of the Yanigua Formation have been studying for many years^25,28–32,38,42,67^. Based on these results, following depositional systems of the Yanigua complex have been proposed: (1) lagoon to coastal^32,41^; (2) shallow water marine to marshy lagoon (with presence of calcarenites containing large number of fauna species, oxidised fragments of plants, and transient conditions from anaeorobic to aerobic^25^; (3) transition from lagoon to carbonate shelf sedimentation (the Yanigua Formation turns to the Los Haitites Formation)^25^; and (4) shallow-marine environment separated from the open sea, locally marshy environment and floodplains^31^.

In the most studies, despite the differences in proposed depositional systems, it was concluded that amber was deposited in a low energy^31,42^ and low salinity water environment^67^.

Further constraints on the depositional system of the Yanigua Formation may be revealed on the basis of mineralogical data related to composition and microtextures of ore mineral assemblages. Pyrite is often proposed as a proxy indicator of precipitation environment^68–71^. In this studies, different generations of pyrite were identified based on their specific morphology: framboids, sunflowers, euhedral and detrital grains (Fig. 6A–D,F). The most symptomatic for palaeoenvironmental interpretations is framboidal pyrite because it forms in a multi-step, redox-dependent process^70^ that is linked to syngenetic^72^ or early diagenetic stage of host rock alteration^73^. Framboidal pyrite is formed in course of consecutive reactions in presence of different oxygen concentrations^70^. Thus, the oxic/anoxic boundary provide the most favourable environment for crystallization of framboidal pyrite form. More detailed data on the redox conditions of framboids precipitation pathways might be revealed from distribution pattern of their diameters^74^. In case of sediments from EMD, the large size of framboids (up to 100 µm) and the presence of another forms of pyrite, rather suggest diagenetic origin of this mineral, which was probably formed under oxic or dysoxic water column^75^. However, if framboidal pyrite was formed during pseudomorphic replacement of certain organic compounds, the determination of depositional system may be ambiguous. Thus, alternating anoxic/dysoxic conditions cannot be entirely excluded as evidenced by geochemical *fingerprint* of associated organic matter, especially low Pr/Ph ratio^76,77^ (Table 3). The oxygen depletion may occur periodically as a result of upwelling process, which played an important role during the formation of the YF complex^30^.

The dominant presence of pyrogenic quartz with minor contribution of plagioclases and K-feldspars (orthoclase, sanidine) as well as abundant occurrence of titanium oxides may indicate some volcanic or volcaniclastic rocks of Los Ranchos Formation (e.g. quartz porphyries, tuffs)^78,79^ as a potential source area for clastic material of studied rocks. The exposure of this complex is located about 1 km far from the SC-02 borehole, so the clastic material was then transported over short distance. The presence of volcaniclastic rocks (tuffs) that might provide the chemical constituents necessary for formation of clay minerals such as Ca-Mg smectite. Additionally, monazite [(Ce, La, Nd, Pr, Gd)PO_4_] was determined in studied rocks. This mineral usually occurs in acid, oversaturated igneous rocks and their tuffs, as well as metamorphic rocks such as schist and gneiss. It is very resistant to weathering processes, therefore it is also a common component of sedimentary rocks. Some monazite crystals are regularly washed by the sea waters and accumulate in deltaic, beach or shallow marine sediments^80–84^.

The absence of illite in investigated rocks suggest a very low degree of diagenesis^85,86^. The presence of mirabilite in the rocks also indicates a shallow diagenesis because this fragile mineral is unstable under higher temperatures^87^.

Only small amounts of aromatic compounds were found in the samples, no naphthalene and its derivatives are present, whereas phenanthrene and its derivatives are in low concentrations. Therefore, most of maturation indices cannot be calculated.

However, maturation of organic matter may be indirectly estimated from the formula proposed by Jarvie et al.^88^,

$$\text{Cal}\%\text{VRo}=0.0180\cdot\text{T}_{\max}-7.16;$$

where Cal %VRo—calculated vitrinite reflectance equivalent, T_max_—maximum temperature measured due to the Rock–Eval.

A cal %VRo value of 0.38 (averaged for samples SC1 and SC2) is indicative for very low degree of OM maturation.

### Comparison of Dominican amber-bearing regions

The two mining districts found in north (NMD) and east (EMD) of the Dominican Republic seem to represent one single sedimentary basin of the Pre-Ocean (likely the Caribbean Sea), later disrupted by left lateral and vertical tectonic movements during the Early to Middle Miocene^25,28,38^. Over the time the epeirogenic or orogenic processes triggered the mass wasting, causing the sedimentation of terrigenous (clastic-organic) sediments in nearby basins.

For the rocks of the El Valle area (EMD), the presence of Ca-Mg smectite, carbonates (calcite, aragonite, dolomite) or sulfates (mirabilite) indicates a saltwater environment of deposition of terrigenous material. The reservoir was likely subjected to denudational movements, which promoted cyclic sedimentation processes of plant detritus and resins. This is also supported by the rich fossil contents, mainly dominated by fauna remnants, such as mollusks, ostracods, foraminifera, bryozoans, red algae, echinoids that are commonly present in marine-terrestrial environment. However, the clastic material of rocks probably originated from the volcanic and/or volcaniclastics of the Los Ranchos complex, found in close vicinity of amber-bearing sediments in the El Valle region.

In the case of La Cumbre deposit (NMD), the presence of kaolinite indicates an oxic environment of low pH, resulting from progressive accumulation of organic matter^10^. Framboidal pyrite started to precipitate when the concentration of oxygen was lowered to anoxic conditions. The results of facies and mineralogical analyses of amber-bearing strata suggest that the environment of marine sedimentation was likely transitional between a shallow maritime lake and periodically flooded plain^10^. The clastic material probably came from the uplifted and exposed rocks of the Pedro Garcia complex^10,25–28,37^.

The significant accumulations of plant detritus, derived from the same botanical source, i.e. the *Hymenaea protera* (*Fabaceae*) tree species, are found in the rocks from both mining districts. In the sediments from the La Cumbre (NMD) the plant detritus consists of larger tree fragments, branches and fruits^10^. This material has undergone significant alterations, firstly rotting in a highly oxidised environment, and then the transformation at more reducing conditions. As a result strongly altered sediment with locally occuring characteristic soft lignite was formed^10^. In the case of the rocks from the El Valle (EMD), the nature of the plant detritus is quite different. It is mainly small fragments of plants, leaves and even grasses, well preserved and showing internal structure which is diagnostic for wood. It might be the result of long-term rest in seawater, which has well-known good preservative properties.

Along with the plant detritus provided from the land to the sedimentation basin, amber crumbs originating from tapping trees were also accumulated. The present study and works of others^10,61,89–91^ have shown that microhardness and density values of resins from the Hato Mayor Province (EMD) are slightly higher than those from the Santiago Province (NMD).

Analysis of biomarker provides further information on the conditions of organic matter deposition as well as its genetic source of the rocks from both regions. The primary indexes used in the reconstruction of palaeoenvironment were: *n*-alkanes distribution, CPI, Pr/Ph, TAR_HC_, Pr/*n*-C_17_, Pr/*n*-C_18_, C_27_-C_28_-C_29_ steranes and P_aq._ ratios.

The Carbon Preference Index (CPI) is commonly used for the determination of the source of *n*-alkane and maturity of organic matter^92^. Immature source rocks with significant input of land-plant organic matter are usually dominated by the odd-carbon-numbered *n*-alkanes, particularly *n*-C_27_, *n*-C_29_, and *n*-C_31_. These *n*-alkanes originate from epicuticular waxes and they are either are synthesized directly from higher plants or defunctionalized even-numbered acids, alcohols or esters^93^. In the Siete Cañadas area, the samples from SC-02 borehole have CPI values ranging from 1.26 to 2.67 with the sample SC3 showing the highest value (Table 3). These results suggest that the source rocks had mixed terrestrial/marine organic matter sedimented in anoxic and dysoxic depositional environment^64,94–96^. These values are comparable to *n*-alkane CPIs for the estuary sediments^97^. For the reference, the rocks from the La Cumbre deposit exhibit a slightly higher CPI values (Table 3) that suggest the presence of the mixed origin of organic matter, in large portion of terrestrial material. The environment conditions during deposition were generally more oxic than in Siete Cañadas area.

The properties of the isoprenoids, natural hydrocarbons mainly of plant origin, have been also used in identification of the deposition environment and source of organic matter. The higher concentration of pristane than phytane was observed in the rocks from the Siete Cañadas area. The calculated pristane/phytane (Pr/Ph) ratio was in the range from 0.49 to 0.71 (Table 3). In the organic matter from La Cumbre deposit, the concentration pristine and phytane was almost the same, the calculated ratio was in range from 0.92 to 1.01 (Table 3). In the case of rocks from the Siete Cañadas area, the very low values of the Pr/Ph below 0.8 (Table 3) suggest anoxic/hypersaline or carbonate environments^76^. The values of Pr/Ph in the range of 1.0–2.0 suggest a dysoxic environment^93–95^. The calculated Pr/Ph ratio for rocks from La Cumbre deposit may suggest that organic matter was deposited under transitional, between anoxic and oxic conditions. Hence, it can be concluded that the organic matter was accumulated in both areas mostly under reducing conditions, in the presence of low oxygen concentration.

The terrigenous/aquatic (TAR_HC_)^66^, and P_aq._ ratios^98^, also were used as an indicator of terrigenous and aquatic organic matter components. In samples from Siete Cañadas area, TAR_HC_ ranges from 1.34 (SC1 sample) to 8.56 (SC4 sample) with median of 4.25. TAR_HC_ values 6.05 and 7.04 were observed for samples from La Cumbre deposit. Despite the differences in values of TAR_HC_, the terrigenous component, from debris of higher plant, is clearly visible in both areas. The P_aq._ values fall in the range from 0.19 to 0.38 for rocks from Siete Cañadas area. The lower values, i.e. 0.13 and 0.15, were found for rocks from the La Cumbre deposit (Table 3). Ficken et al.^98^ reported that the P_aq._ values ranging from 0.01 to 0.23 are diagnostic for terrestrial plant waxes, whereas the values in the range 0.48–0.94 for submerged/floating macrophytes. The results obtained for Siete Cañadas area suggest the dominance of submerged/floating macrophytes, whereas P_aq values_ for rocks from the La Cumbre deposit indicate the higher plant/macrophyte waxes.

**Figure 11:**
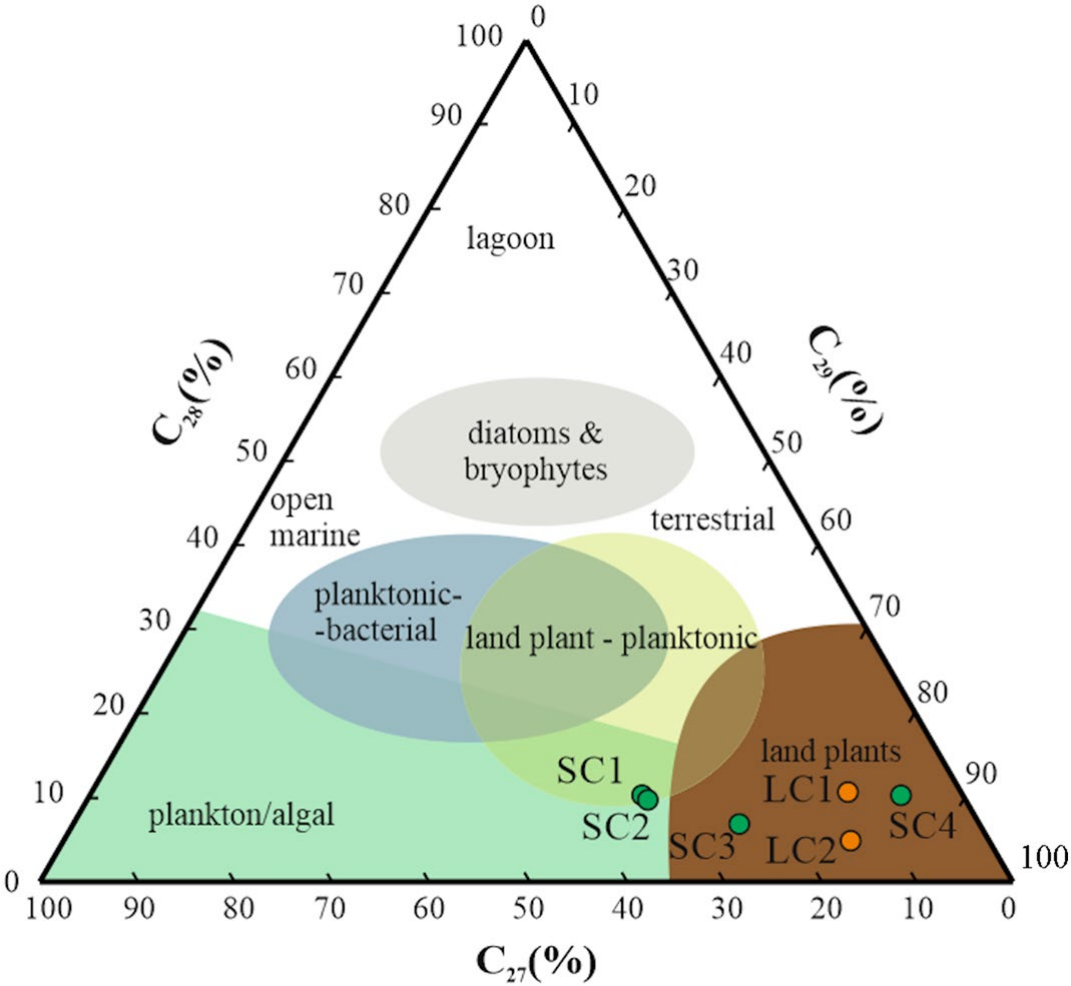
A ternary plot of C_27_ vs. C_28_ vs. C_29_ steranes (as normalised percentages; after Huang and Meinschein^99^, Peters et al.^93^, modified).

C_27_, C_28_ and C_29_ steranes from both regions have similar distributions (C_29_ > C_27_ > C_28_) (Table 4), and indicate a terrestrial source of organic matter (Fig. 11). Only in the samples SC1 and SC2 from the Siete Cañadas, higher amounts of pentacyclic terpanes were found which suggests presence of plankton/algal organic matter fraction.

## Conclusions

The results of mineralogical and biomarker analyses of amber-bearing mudstones collected from drill holes in Eastern Mining District were discussed and compared with data obtained for amber-bearing coaly shales found in the second mining district in the north of the Dominican Republic (NMD). The major conclusions are as follows:

1. The source area of the clastic material for the La Cumbre deposit (NMD) probably was the Pedro Garcia complex of granitoids and acid pyroclastic rocks. In the case of the Siete Cañadas zone (EMD) the clastic material probably originated from the erosion of volcanic and/or volcaniclastic rocks of the Los Ranchos complex. In both areas, the clastic material was dominated by clay minerals, quartz, feldspars and subordinate mica, amphibole-group minerals, zircon, anatase, rutile, hematite, ilmenite.
2. The deposition of the clastic material in both areas proceeded in marine environment. In northern district, the sedimentation was in the lagoon environment, a shallow maritime lake or periodically flooded plain. In eastern district the deposition probably took place in a shallow-marine basin affected by the denudation processes.
3. Amber is hosted in organic substance-bearing mudstones in Siete Cañadas (EMD) and coaly shales and lignite in La Cumbre (NMD). In the eastern region, the immature organic matter found in sediments has mixed terrestrial/marine origin and was deposited in anoxic and dysoxic depositional environment. In northern area more matured organic substance is also of mixed origin, but with a greater proportion of terrestrial material. It was accumulated under more oxic palaeo-conditions.

Palaeoenvironmental reconstruction requires broad interdisciplinary studies. Further, additional stratigraphic, sedimentological, geochemical and palaeontological studies are required to specify in more detail the formation environment of the amber -bearing deposits in the Dominican Republic. Some palaeoenvironmental fluctuations can be reconstructed using qualitative and quantitative palaeontological analyses of fossils with carbonate shells supported by the determination of trace elements (Mg/Ca) and stable isotopes (δ^18^O and δ^13^C) in the carbonates. In addition, the redox conditions can be elucidated by the determination of redox-sensitive trace metals in sediments. The application of various geothermometers such as coral growth bands, clay-mineral thermometry or Raman-based carbonaceous material thermometry may also helpful in elucidation of palaeoenvironment conditions. Hence, aforementioned interdisciplinary studies will be a subject of future work. The results will contribute to the development of research into the unique properties of Dominican ambers.

## Data Availability

The datasets generated during and/or analysed during the current study are available in the Mendeley Data repository, http://dx.doi.org/10.17632/ydrbh8b33f.1.
